# Investigating the association between cholinergic integrity and amyloid burden using cholinergic anatomy and [^18^F]‐FEOBV PET in individuals with Down Syndrome from the ABC‐DS and TRC‐DS cohorts

**DOI:** 10.1002/alz.091823

**Published:** 2025-01-09

**Authors:** Jason K Russell, Alexander C Conley, Brian Boyd, Rachel Schlossberg, Allison Stranick, Adam J Rosenberg, Lealani Mae Y. Acosta, Michael Rafii, Sepideh Shokouhi, Paul A Newhouse

**Affiliations:** ^1^ Center for Cognitive Medicine, Vanderbilt University Medical Center, Nashville, TN USA; ^2^ Vanderbilt University Medical Center, Nashville, TN USA; ^3^ Alzheimer's Therapeutic Research Institute, University of Southern California, San Diego, CA USA; ^4^ GRECC, VA, Tennessee Valley Healthcare System, Nashville, TN USA

## Abstract

**Background:**

Adults with Down Syndrome (DS) have the highest risk of developing Alzheimer’s disease (AD) worldwide. Triplication of the amyloid precursor protein gene on chromosome 21 results in early amyloid accumulation and Alzheimer’s pathology. The cholinergic system is known to decline early in the development of AD and plays a fundamental role in observed cognitive deficits. In the present work, we assess the association between amyloid accumulation and cholinergic integrity.

**Method:**

188 individuals from the ABC‐DS cohort (U01AG051406; U01AG051412; U19AG068054) were assessed cross‐sectionally. Basal forebrain volumes were calculated using the ScLimbic FreeSurfer pipeline for the basal forebrain (Ch1‐Ch4) and the DnSeg pipeline for Ch4 volumes. Linear regressions were performed between centiloid values, age, and basal forebrain volumes. Nine individuals recruited for a TRC‐DS sub‐study underwent a multimodal imaging assessment, including [^11^C]‐PiB PET and [^18^F]‐FEOBV PET imaging. Global centiloid values were calculated using PMOD, with regional SUVRs calculated using PetSurfer with GTM for partial volume corrections. Linear regressions were performed between global centiloid, regional [^11^C]‐PiB SUVR, and regional [^18^F]‐FEOBV SUVR.

**Result:**

In ABC‐DS and the TRC‐DS sub‐study, global amyloid accumulation was positively associated with age (p<0.0001 and p=0.041). Age and global amyloid accumulation were negatively associated with Ch1‐Ch4 (p<0.005) and Ch4 (p<0.05) cholinergic basal forebrain volumes. In the TRC‐DS sub‐study, global amyloid accumulation negatively associated with [^18^F]‐FEOBV uptake in the amygdala (p<0.05), statistical trends were observed in the thalamus and left entorhinal cortex (p<0.08). Regional [^11^C]‐PiB uptake was negatively associated with [^18^F]‐FEOBV uptake in the left inferior temporal and right pericalcarine cortices (p<0.05), with statistical trends observed in the right amygdala, left pericalcarine, and left fusiform cortices (p<0.08).

**Conclusion:**

Measures of cholinergic integrity were found to decline with increasing amyloid in individuals with DS across two cohorts, utilizing different methodologies. In the ABC‐DS cohort, we observed a reduction in basal forebrain nuclei volume with increasing age and amyloid accumulation. The basal forebrain contains cholinergic and noncholinergic neurons. To understand whether this volume reduction was from cholinergic loss, we assessed the integrity of cholinergic terminals using [^18^F]‐FEOBV PET and found regional cholinergic integrity negatively associated with both global and regional amyloid accumulation.

